# Phytochemical Content, Antioxidant and Antimicrobial Potential of *Althaea officinalis* L. Extracts Prepared by “Green” Classical and Natural Deep Eutectic Solvents

**DOI:** 10.3390/molecules31101575

**Published:** 2026-05-08

**Authors:** Neli Memdueva, Milena Tzanova, Plamena Staleva, Mariana Kamenova-Nacheva, Kalina Krastilova, Zvezdelina Yaneva, Nikolina Rusenova, Neli Grozeva, Stela Ginin, Toncho Dinev

**Affiliations:** 1 Department of Biological Sciences, Faculty of Agriculture, Trakia University, 6000 Stara Zagora, Bulgaria; neli.memdueva.21@trakia-uni.bg (N.M.); n.grozeva@traki-uni.bg (N.G.); stela.ginin@trakia-uni.bg (S.G.); 2 Research and Development and Innovation Consortium, Sofia Tech Park JSC, 111 Tsarigradsko Shose Blvd., 1784 Sofia, Bulgaria; plamena.staleva@orgchm.bas.bg (P.S.); mariana.nacheva@orgchm.bas.bg (M.K.-N.); 3 Institute of Organic Chemistry with Centre of Phytochemistry, Bulgarian Academy of Sciences, Acad. G. Bonchev Str. 9, 1113 Sofia, Bulgaria; 4 Centre of Competence “Sustainable Utilization of Bio-Resources and Waste of Medicinal and Aromatic Plants for Innovative Bioactive Products” (BIORESOURCES BG), 1000 Sofia, Bulgaria; kalina.krastilova@orgchm.bas.bg; 5 Department of Pharmacology, Animal Physiology, Biochemistry and Chemistry, Faculty of Veterinary Medicine, Trakia University, 6000 Stara Zagora, Bulgaria; zvezdelina.yaneva@trakia-uni.bg; 6 Department of Veterinary Microbiology, Infectious and Parasitic Diseases, Faculty of Veterinary Medicine, Trakia University, 6000 Stara Zagora, Bulgaria; n_v_n_v@abv.bg

**Keywords:** marshmallow, ethanol, natural deep eutectic solvents, HPLC-PDA-MS, HPLC-HRMS/MS, GC-MS, antimicrobial activity, antioxidants

## Abstract

Due to its abundant content of biologically active compounds, *Althaea officinalis* L. (marshmallow) has been extensively researched and applied in both the food and pharmaceutical industries. This study aimed to evaluate hydroethanolic and natural deep eutectic solvent (NADES) extracts from leaves, flowers, and roots in terms of their chemical composition and biological activities. Extracts were characterized using chromatographic and spectrophotometric methods. Phytochemical profiling by HPLC-MS revealed a diverse composition, with 35 secondary metabolites identified or tentatively assigned, mainly hydroxycinnamic acid derivatives and flavonoid glycosides. GC-MS analysis of the ethanol extracts identified ten free amino acids, seven organic acids, several mono- and disaccharides, and one oligosaccharide. Their concentrations varied across different parts of the plant depending on the specific metabolism of the respective organ. Ethanolic extracts showed the highest total phenolic content (up to 176 mg GAE/L), while flower extracts exhibited the strongest antioxidant activity (DPPH up to 89 µmol TE/L). The antimicrobial potential of the extracts was assessed by the agar well diffusion method. NADES1 extracts demonstrated significant antibacterial activity, with inhibition zones reaching up to ~34 mm, whereas NADES2 extracts were largely inactive. In contrast, antifungal activity was negligible or absent across all samples.

## 1. Introduction

Medicinal plants are a rich source of bioactive compounds, resulting in diverse therapeutic properties, with many species exhibiting a predominant pharmacological activity that defines their primary medicinal effect. These plants are traditionally used individually or in herbal formulations, and their bioactive constituents are often isolated for the development of pharmaceutical preparations with a wide range of activities, including antimicrobial activity against multidrug-resistant microorganisms [1,2,3]. Cultivation through agrobiological techniques and selective breeding enables the production of varieties with high concentrations of active compounds, vitamins, enzymes, and microelements, ensuring consistent quality, whereas wild species show substantial variability in bioactive content [4,5].

In recent decades, botanical and pharmacological research on wild plants worldwide has expanded; however, the chemical composition of many medicinal species remains insufficiently investigated [6]. *Althaea officinalis* L. (known as “marshmallow”) is a perennial herbaceous plant native to Eastern Europe and Western Asia, belonging to the Malvaceae family [7]. The plant’s roots and leaves are edible and used in teas and salads [8,9]. Historically, the entire plant has been recognized for its medicinal properties, including treatment of sore throat, dry cough, gastric ulcers, wounds, burns, insect bites, rhinitis, abscesses, constipation, and diarrhea [9,10]. Contemporary studies demonstrate that *A. officinalis* extracts exhibit antitussive, anti-inflammatory, antioxidant, antibacterial, antiulcer, wound-healing, and immunomodulatory activities [7,11,12]. Beyond therapeutic applications, *A. officinalis* has been utilized in the food industry; notably, the marshmallow confection, which has been consumed worldwide for over 4000 years, was initially formulated as a cough remedy [13].

Modern extraction methodologies prioritize environmental sustainability, with green solvents playing a pivotal role [14]. When these green solvents are both efficient and selective, they can lead to a considerable decrease in production expenses [15]. Natural deep eutectic solvents (NADESs), comprising hydrogen bond donors (HBDs) and acceptors (HBAs) derived from naturally occurring compounds [16], offer low melting points, non-toxicity, renewability, cost-effectiveness, and ease of preparation [17,18]. Nevertheless, their high viscosity limits their use, as it hinders mass transfer and complicates extraction. The addition of water up to 30% can improve fluidity, but this may reduce extraction efficiency for non-polar compounds as well [15]. Choline chloride-based NADESs are particularly advantageous due to their accessibility, stability under humid conditions, non-toxicity, and alignment with green chemistry principles [19,20]. These solvents facilitate phase separation, allowing solvent isolation through simple decantation [21], and provide high extraction capacity for polyphenolic compounds, which are recognized for strong antioxidant activity [16,22]. Compared to conventional solvents, NADESs often demonstrate superior polyphenolic extraction, attributed to strong intermolecular interactions between the solvent and the dissolved compounds [23], and yield extracts with enhanced antioxidant potential [18,24]. Additionally, NADES extracts can be applied directly in the food sector [18].

According to the scientific literature, *A. officinalis* has been most extensively studied in samples of Asian origin, including Iran [10,25,26,27,28,29], China [12,30,31], Lebanon [8,32], and India [33,34], whereas fewer studies have focused on European samples, such as those from France [35], Germany [36,37], Poland [38], and Bulgaria [39,40]. A review by Baimakhanova et al. (2025) highlighted that leaves and roots are the most frequently examined plant parts, while water and alcoholic solvents (methanol and ethanol) are the most frequently used for extract preparation [9]. Although it is commonly utilized, the chemical composition of *A. officinalis* has not been comprehensively characterized, and existing studies provide only partial and fragmented phytochemical data. In this context, the objective of the current research was to assess the potential of NADESs as green alternatives to classical ethanol for the extraction of bioactive substances from different plant organs of *Althaea officinalis* and to characterize the resulting extracts in terms of their phytochemical composition and antioxidant, antibacterial, and antifungal activities.

## 2. Results and Discussion

Marshmallow contains natural ingredients recognized for their antioxidant properties, e.g., phenols, flavonoids, fatty acids, and alkaloids [8,30,31,41]. Depending on the selected solvent, the extraction method utilized, and the plant organ chosen, one group of these biologically active substances can be selectively extracted. For instance, Moazzezi et al. acquired a mucilage-rich extract from seeds by maceration in water at a ratio of 50:1 *v*/*w* at room temperature [29]. Water was the right choice for mucilage extraction from marshmallow roots by other research teams as well [12]. The authors found that the significant antioxidant activity of the extracts resulted from the elevated levels of flavonoids, phenolic acids, scopoletin, phytosterols, amino acids, tannins, coumarins, asparagine and glycine betaine. In contrast, a hydroethanolic solvent had a stronger capacity to extract antioxidant constituents from *A. officinalis* roots than water [39].

In the quest for environmentally friendly solvents, our research team chose two natural deep eutectic solvents utilizing choline chloride (ChCl) as HBA along with citric acid (CA) or glycerol (Gly) as HBD. This choice was based on their demonstrated capacity to extract polyphenols. The extracts derived have been shown to frequently exhibit greater antioxidant potential [15,42,43]. In the current research, their capability to extract antioxidants from various plant organs of marshmallow was compared to the “green” classical ethanol.

To evaluate the extraction efficiency, three distinct plant organs of *A. officinalis* (leaves, flowers, and roots) were treated with two different NADES formulations and a conventional solvent (70% ethanol) for comparison. The experimental design is summarized in Table 1.

### 2.1. Chemical Profiling and Antioxidant Potential of A. officinalis Extracts

#### 2.1.1. Phytochemical Profiling by LC–MS

Comprehensive phytochemical profiling of extracts obtained from the leaves, flowers, and roots of *Althaea officinalis*, prepared using natural deep eutectic solvents and hydroethanol, revealed a chemically diverse composition dominated by flavonoid glycosides and hydroxycinnamic acid derivatives. Despite its traditional medicinal use, the phytochemical characterization of *A. officinalis* remains limited, and the present study substantially expands the currently available data. A total of 35 secondary metabolites were detected using HPLC-PDA-MS and HPLC-HRMS/MS analyses (Table 2), highlighting the chemical complexity of this species. HPLCHRMS/MS analysis was performed only for hydroethanolic extracts to support compound identification, whereas NADES extracts were characterized primarily using HPLC-PDA-MS. Compound identification was based on accurate mass measurements, MS/MS fragmentation patterns, UV–VIS spectra, and comparison with literature data for *A. officinalis* and related species within the *Malvaceae* family [37,44,45,46,47,48,49,50], as well as online databases [51].

**Figure 1:**
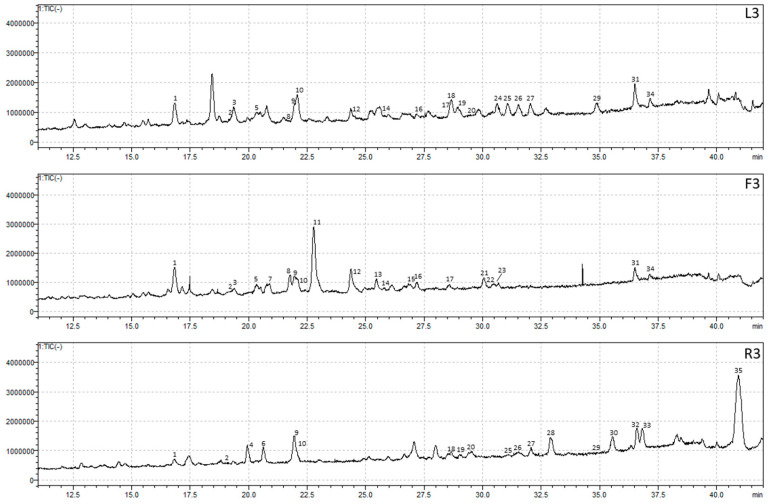
Total ion current (TIC) chromatogram obtained by HPLC-PDA-MS analysis of hydroethanolic extracts L3, F3, and R3. Numbers indicate the identified chromatographic peaks corresponding to the compounds listed in Table 2.

**Figure 2:**
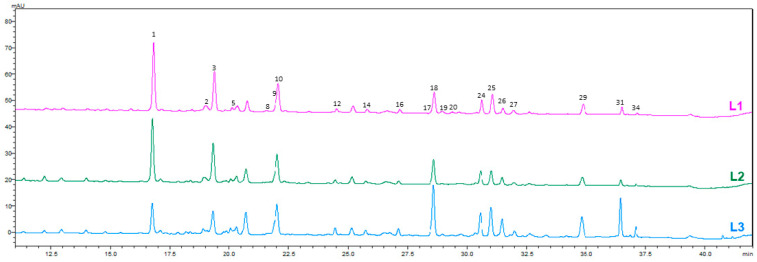
HPLC–PDA chromatograms of hydroethanolic and NADES extracts of leaves of *A. officinalis* at 350 nm. Numbers indicate the identified chromatographic peaks corresponding to the compounds listed in Table 2.

**Figure 3:**
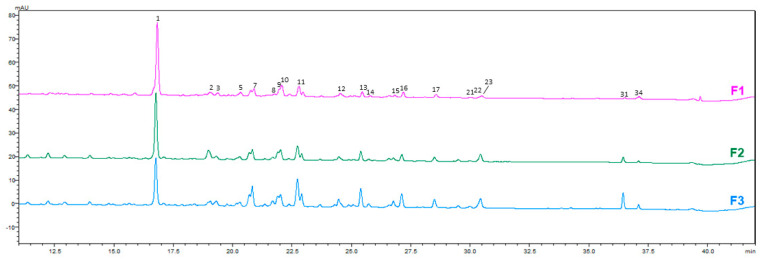
HPLC−PDA chromatograms of hydroethanolic and NADES extracts of flowers of *A. officinalis* at 350 nm. Numbers indicate the identified chromatographic peaks corresponding to the compounds listed in Table 2.

**Figure 4:**
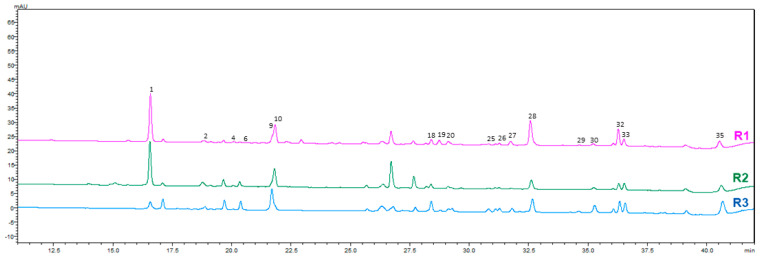
HPLC−PDA chromatograms of hydroethanolic and NADES extracts of roots of *A. officinalis* at 350 nm. Numbers indicate the identified chromatographic peaks corresponding to the compounds listed in Table 2.

Seven compounds were confirmed using authentic standards, including caffeic acid, rutin, quercetin 3-glucoside, kaempferol 3-rutinoside, kaempferol 3-glucoside, and cis- and trans-tiliroside. The remaining metabolites were tentatively assigned based on spectral characteristics and fragmentation patterns. The comparative HPLC-PDA chromatograms of *A. officinalis* leaf, flower, and root extracts obtained using three different solvents are shown in Figure 1, Figure 2, Figure 3 and Figure 4.

Four metabolites were detected across all plant parts, including 2-caffeoylhydroxycitric acid (**1**), caffeic acid (**2**), N-(E)-caffeoyl-l-dopa (**9**), and 2-*O*-feruloylhydroxycitric acid (**10**). These compounds belong primarily to hydroxycinnamic acid derivatives, which were broadly distributed across the studied plant organs. Additionally, 2-caffeoylisocitric acid (**3**) and 2-O-p-coumaroylhydroxycitric acid (**5**) were detected in leaves and flowers but were absent in root extracts.

Other organ-dependent differences were also observed. Leaves and flowers were characterized by a higher abundance of quercetin- and kaempferol-based glycosides, including rutin (**12**), quercetin 3-glucoside (**14**), kaempferol 3-rutinoside (**16**), kaempferol 3-glucoside (**17**), and *cis*- and *trans*-tiliroside (**34**, **31**), as well as flavonoid diglycoside II (**8**). Additionally, seven metabolites were detected only in flower extracts, including dihydrokaempferol-7-*O*-β-d-glucopyranoside (**11**), naringenin 7-*O*-β-d-glucoside (**21**), kaempferol malonyl glycoside (**22**), kaempferol glucopyranoside (**23**), and other flavonoid glycosides (**7**, **13**, **15**), highlighting the distinct phytochemical contribution of floral tissues. In contrast, a single compound, putatively assigned as quercetin malonyl glycoside (**24**), was detected exclusively in leaf extracts.

Root extracts exhibited a distinct profile characterized by sulfated flavonoid derivatives and hypolaetin-related compounds, which closely aligns with the findings reported by other authors too [37]. These included flavonoid *O*-sulfoglycosides (**4**, **6**), hypolaetin-8-*O*-β-d-(3″-*O*-sulfo)glucuronopyranoside (**28**), 4′-*O*-methylhypolaetin-8-*O*-β-d-(3″-*O*-sulfo)glucuronopyranoside (**32**), isoscutellarein 8-*O*-β-glucuronopyranoside 3″-*O*-sulfate (**33**), and 4′-*O*-methylisoscutellarein-8-*O*-β-d-(3″-*O*-sulfo)glucuronopyranoside (**35**), which were detected exclusively in roots. Compound **30** (isoscutellarein derivative) was predominantly observed in roots and detected only in trace amounts in leaves.

**Table 2:** Data from the HPLC-PDA-MS and HPLC-HRMS/MS analysis of hydroethanolic and NADES extracts of leaves, flowers, and roots of *A. officinalis*.

No	RT ^a^ min	RT ^b^ min	Compound	UV ^a^ λmax, nm	[M−H]^− b^	Δ ^b^, ppm	Formula	MS/MS Fragments ^b^	L1	L2	L3	F1	F2	F3	R1	R2	R3	Ref.
1	16.91	6.07	2-Caffeloylhydroxycitric acid	236, 330	369.0464	0.66	C_15_H_14_O_11_	207, 189, 127, 83	+	+	+	+	+	+	+	+	+	[44]
2	19.06	5.90	Caffeic acid	239, 330	179.0340	5.28	C_9_H_8_O_4_	179, 135	+	+	+	+	+	+	+	+	+	Std
3	19.41	7.52	2-Caffeloylisocitric acid or isomer	239, 330	353.0518	1.00	C_15_H_14_O_10_	191, 173, 111	+	+	+	+	+	+	−	−	−	
4	19.97	9.36	Flavonoid *O*-sulfoglycoside I	240, 273, 330	543.0456	1.07	C_21_H_20_O_15_S	543, 301, 241, 133	−	−	−	−	−	−	+	+	+	
5	20.31	8.18	2-*O*-Coumaroylhydroxycitric acid	238, 315	353.0517	0.84	C_15_H_14_O_10_	207, 189, 127, 83	+	+	+	+	+	+	−	−	−	[51]
6	20.56	10.06	Flavonoid *O*-sulfoglycoside II	241, 273, 330	557.0611	0.56	C_22_H_22_O_15_S	557, 477, 315, 300, 241, 137	−	−	−	−	−	−	+	+	+	
7	20.89	10.12	Flavonoid diglycoside I	269, 355	655.1526	1.60	C_28_H_32_O_18_	655, 330, 315, 287	−	−	−	+	+	+	−	−	−	
8	21.75	9.07	Flavonoid diglycoside II	231, 289	465.1039	0.25	C_21_H_22_O_12_	465, 303, 285, 125	+	+	+	+	+	+	−	−	−	
9	21.86	10.03	*N*-(*E*)-caffeoyl-l-dopa	238, 321	358.0936	1.07	C_18_H_17_NO_7_	358, 222,178, 135	+	+	+	+	+	+	+	+	+	[37]
10	22.05	9.54	2-*O*-Feruloylhydroxycitric acid	242, 330	383.0623	0.82	C_16_H_16_O_11_	189, 127, 83	+	+	+	+	+	+	+	+	+	[51]
11	22.78	9.69	Dihydrokaempferol-7-*O*-β-d-glucopyranoside	224, 291	449.1094	0.98	C_21_H_22_O_11_	449, 287, 259, 125	−	−	−	+	+	+	−	−	−	[45]
12	24.53	12.32	Rutin	254, 355	609.1470	1.45	C_27_H_30_O_16_	609, 300, 271, 255	+	+	+	+	+	+	−	−	−	Std
13	25.45	12.77	Flavonoid diglycoside III	264, 347	579.1362	0.75	C_26_H_28_O_15_	579, 284, 255, 227	−	−	−	+	+	+	−	−	−	
14	25.77	12.65	Quercetin 3-glucoside	246, 352	463.0890	1.64	C_21_H_20_O_12_	463, 300, 271, 255	+	+	+	+	+	+	−	−	−	Std
15	26.89	14.49	Flavonoid triglycoside	244, 272	767.2049	1.28	C_34_H_40_O_10_	665, 623, 314, 299, 271	−	−	−	+	+	+	−	−	−	
16	27.26	14.13	Kaempferol 3-rutinoside	245, 350	593.1520	1.28	C_27_H_30_O_15_	593, 285, 284, 255, 227	+	+	+	+	+	+	−	−	−	Std
17	28.61	14.50	Kaempferol 3-glucoside	264, 346	447.0938	1.13	C_21_H_20_O_11_	447, 285, 284, 255, 227	+	+	+	+	+	+	−	−	−	Std
18	28.65	14.96	Hypolaetin-8-*O*-β-d-glucopyranosyl-(1‴ → 4″)-β-d-glucuronopyranoside	268, 352	639.1284	1.36	C_27_H_28_O_18_	639, 459, 371, 301, 133	+	+	+	−	−	−	+	+	+	[37]
19	29.01	14.54	Hypolaetin-8-*O*-d-glucoside-3′-sulfate	246, 350	543.0457	1.24	C_21_H_20_O_15_S	543, 301, 255, 133	+	+	+	−	−	−	+	+	+	[46,50]
20	29.41	15.22	Hypolaetin-8-β-d-glucuronopyranoside	246, 311	477.0680	1.20	C_21_H_18_O_13_	477, 301, 255, 133	+	+	+	−	−	−	+	+	+	[47,50]
21	30.05	14.97	Naringenin 7-*O*-β-d-glucoside (Prunin)	241, 289	433.1143	0.53	C_21_H_22_O_10_	433, 271, 151	−	−	−	+	+	+	−	−	−	[45]
22	30.41	15.98	Kaempferol malonyl glycoside	247, 355	533.0942	1.05	C_24_H_22_O_14_	489, 285, 255, 227	−	−	−	+	+	+	−	−	−	[51]
23	30.51	15.54	Kaempferol glucopyranoside	265, 363	447.0937	0.98	C_22_H_20_O_11_	447, 284, 255, 151	−	−	−	+	+	+	−	−	−	[45]
24	30.64	16.43	Quercetin malonyl glycoside	252, 347	549.0893	1.28	C_24_H_22_O_15_	301, 133	+	+	+	−	−	−	−	−	−	[51]
25	31.06	16.41	Quercitin-3-*O*-β-d-glucopyranoside	269, 351	463.0888	1.27	C_21_H_20_O_12_	301, 133	+	+	+	−	−	−	+	+	+	[48]
26	31.53	17.20	Flavonoid diglycoside IV	252, 349	653.1369	1.40	C_28_H_30_O_18_	653, 473, 429, 315, 300	+	+	+	−	−	−	+	+	+	
27	32.08	16.66	4′-O-Methylhypolaetin-8-*O*-β-d-(2″-*O*-sulfo)glucopyranoside	247, 349	557.0611	0.83	C_22_H_22_O_15_S	557, 477, 315, 300, 241	+	+	+	−	−	−	+	+	+	[37]
28	32.9	17.13	Hypolaetin-8-*O*-β-d-(3″-*O*-sulfo)glucuronopyranoside	253, 352	557.0251	1.39	C_21_H_18_O_16_S	557, 477, 301, 255	−	−	−	−	−	−	+	+	+	[37]
29	34.91	19.03	Flavonoid diglycoside V	251, 347	477.1043	0.96	C_22_H_22_O_12_	477, 315, 300	+	+	+	−	−	−	tr	tr	tr	
30	35.44	18.83	Isoscutellarein 8-*O*-β-glucuronopyranoside 3′′-*O*-sulfate or isomer	245, 272, 330	541.0665	1.35	C_22_H_22_O_14_S	541, 461, 299, 284, 241	tr	tr	tr	−	−	−	+	+	+	
31	36.5	20.58	*trans*-Tiliroside	266, 314	593.1308	1.20	C_30_H_26_O_13_	593, 285, 284, 255, 227	+	+	+	+	+	+	−	−	−	Std
32	36.6	19.62	4′-O-Methylhypolaetin-8-*O*-β-d-(3″-*O*-sulfo)glucuronopyranoside	249, 349	571.0406	1.24	C_22_H_20_O_16_S	571, 315, 300, 255	−	−	−	−	−	−	+	+	+	[37]
33	36.87	19.76	Isoscutellarein 8-*O*-β-glucuronopyranoside 3″-*O*-sulfate or isomer	270, 340	541.0300	1.18	C_21_H_18_O_15_S	541, 461, 299, 285, 255	−	−	−	−	−	−	+	+	+	[49]
34	37.19	21.45	*cis*-Tiliroside	266, 312	593.1309	1.38	C_30_H_26_O_13_	593, 285, 284, 255, 227	+	+	+	+	+	+	−	−	−	Std
35	40.97	24.04	4′-*O*-Methylisoscutellarein-8-*O*-β-d-(3″-*O*-sulfo)glucuronopyranoside	273, 330	555.0457	1.20	C_22_H_20_O_15_S	555, 475, 299, 284, 254, 175	−	−	−	−	−	−	+	+	+	[37]

^a^ Retention time and UV spectra from HPLC-PDA-MS. ^b^ Retention time, *m*/*z*, ∆, formula, and MS/MS fragmentation from UHPLC-HRMS/MS. tr—traces; Ref.—references; Std—standard; “+”—presence; “−”—absence.

Furthermore, several hypolaetin derivatives, including hypolaetin-8-*O*-β-d-glucopyranosyl-(1‴ → 4″)-β-d-glucuronopyranoside (**18**), hypolaetin-8-*O*-d-glucoside-3′-sulfate (**19**), and hypolaetin-8-β-d-glucuronopyranoside (**20**), were detected in both leaves and roots but were absent in flower extracts. Similarly, compounds **25**–**27** and **29** were primarily associated with leaves and roots, with compound **29** detected only in trace amounts in root extracts.

Despite these plant part-specific features, solvent-dependent differences were limited. NADES1, NADES2, and ethanolic extracts exhibited largely comparable qualitative profiles, with minor variations mainly observed in peak intensities. These findings indicate similar extraction capabilities across solvent systems and suggest that natural deep eutectic solvents effectively recover a broad spectrum of phenolic compounds.

Overall, the phytochemical profiles of *A. officinalis* extracts revealed both shared and organ-specific features, with clear differences in metabolite distribution among leaves, flowers, and roots. Importantly, NADESs demonstrated metabolite coverage comparable to conventional hydroethanol, supporting their application as environmentally friendly alternatives for the extraction of phenolic compounds from *A. officinalis*.

#### 2.1.2. Primary Metabolite Profiling by GC–MS

GC–MS profiling of *A. officinalis* 70% ethanol extracts from leaves, flowers, and roots confirmed distinct variations in metabolite distribution across the plant organs, too. Three groups of biologically active substances were determined: amino acids, organic acids, and mono- and disaccharides (Table 3).

Key biological activities of marshmallow have been linked to the presence of specific amino acids, e.g., glutamic acid, which has wound-healing and anti-inflammatory effects, and valine and leucine, which possess anti-inflammatory, skin-protective, and respiratory-protective effects [52]. Ten free amino acids were identified in all tested organs: glutamic acid, aspartic acid, alanine, serine, glycine, proline, asparagine, valine, leucine, and phenylalanine. The flowers were characterized by the largest total amount (28.7 mg/g dm) and the roots by the smallest amount of these compounds (12.9 mg/g dm). The leaf extracts showed the highest content of glutamic acid (4.1 ± 0.9 mg/g dm) and aspartic acid (3.6 ± 0.8 mg/g dm); the flower showed the highest content of proline (4.5 ± 1.1 mg/g dm); and the roots showed the highest content of asparagine (5.6 ± 1.4 mg/g dm). Alanine, serine, glycine, valine, leucine, and phenylalanine had similar quantity distribution in leaves and flowers (Table 3). In other studies, the identified free amino acids were the same, with only the quantities differing. Leaves contained the highest levels of glutamic acid, glycine, aspartic acid, and alanine [53,54], while in roots, aspartic acid was prevalent, followed by arginine [55], and alanine, aspartate, glutamate, and glycine in similar quantities [54].

The group of organic acids, determined in the present study by the GC-MS method, included seven compounds: malic, citric, oxalic, succinic, fumaric, galacturonic and glucuronic acids (Table 3), which are metabolites of high importance. For example, malate, citrate, succinate, and fumarate are present in the Krebs cycle. The glucose derivatives offer significant health benefits: galacturonic acid acts as a probiotic and promotes gut health [56], while glucuronic acid conjugates with toxic substances and plays a leading role in detoxification [57]. In the present study, the total content of organic acids in the leaves was the highest (29.5 mg/g dm), while that in the roots was the lowest (17.5 mg/g dm). In the aerial plant organs, Krebs cycle metabolites predominated, while in the roots, glucose derivatives dominated. In the flower extracts no glucuronic or galacturonic acids were detected (Table 3). Also, D-glucuronic and D-galacturonic acids were identified in the aqueous extracts of the roots [9].

According to the GC-MS analysis, 70% ethanol also extracted four monosaccharides (glucose, fructose, arabinose, and rhamnose), three disaccharides (sucrose, maltose, and galactose), and one oligosaccharide (raffinose), which were present in varying quantities in different plant organs (Table 3). According to many studies, the roots are rich in polysaccharides (20–35%) [52]. In contrast, in the present study, flowers showed the largest total amount of mono- and oligosaccharides (201.9 ± 29.7 mg/g dm), which was almost twofold higher than those in the roots and leaves. Arabinose, galactose, and rhamnose were detected only in the roots, and maltose was detected only in the aerial plant parts. Other researchers have also found galactose and rhamnose in roots extracted by water [9]. Using GC-MS analysis, Farhat et al. demonstrated the presence of mono- and disaccharides, such as ribose, arabinose, fructose, and galactose, in aqueous flower extract [8]. Some mucilaginous mono- and polysaccharides, such as galactose, rhamnogalacturonan, and arabinogalactan, have been shown to be effective in cough treatment [58].

#### 2.1.3. Phenolic Content and Antioxidant Activity

The pH values and all assessed total levels of biological active compounds are summarized in Table 4. The values are provided as equivalents per 1 L of the prepared extract, allowing easier comparison among all extracts obtained in our study.

The extracts with NADES1 exhibited extremely low pH values—from pH = 0.68 ± 0.02 for R1 to pH = 0.96 ± 0.02 for F1. The pH of the pure NADES1 was −0.17 ± 0.02, which explains the exceptionally high acidity of the extracts of this group. Our team received comparable results in a previous investigation [50,59]. NADES2 demonstrated a higher pH = 2.63 ± 0.02, and the extracts derived from it exhibited closely matching pH values: 4.51 ± 0.02, 4.83 ± 0.02, and 4.85 ± 0.02 for roots, leaves, and flowers, respectively.

As expected, different TPC, TFC, TCT and TAntC extraction results were achieved for different plant parts and different solvents (Table 4). The highest TPC, TCT, and TAntC levels were obtained for the ethanolic extracts: TPC ranged from 32 ± 1 mgGAE/L (R3) to 176 ± 4 mgGAE/L (F3); TCT ranged from 16 ± 4 in the roots to 73 ± 6 mgCE/L in the flowers; and TAntC ranged from not detected (R3) to 7.34 ± 0.12 mgCE/L (F3). NADES1 extracted the highest amount of flavonoids from flowers (109 ± 9 mgCE/L). Anthocyanins were not detected in NADES2 extracts or in all root extracts. Anthocyanins were extracted from the flowers by NADES1 at a concentration of 0.29 ± 0.03 mgCGE/L. Similar results were obtained by our research team in studies on *M. sylvestris* [59] and *M. neglecta* [50]. The highest content of these antioxidants (phenolic compounds, flavonoids, condensed tannins and anthocyanins) was found in the extracts from flowers, and the lowest was found in the roots.

Khodadadi et al. compared the potential of a deep eutectic solvent based on ammonium acetate and lactic acid (at a ratio of 1:2) with methanol and water. The authors found twofold higher total phenolic content in DES extracts than in aqueous and methanolic extracts [25]. The extraction of plant metabolites by NADESs relies on their polarity, viscosity, and pH. NADESs exhibiting high viscosity (such as ChCl/CA and ChCl/Gly) were characterized by lower extraction efficiency for phenolic compounds relative to the less viscous deep eutectic solvents [60,61]. Reducing viscosity results in intensification of the cavitation phenomena, leading to enhanced H-bonding between the eutectic solvent and solute, thereby enhancing the extraction efficiency and yield [62].

The differences in the content of the determined active compounds are not solely attributable to the solvents: the reasons are complex. In the search for the biological activities of extracts from medicinal rose, many research teams have also determined the total content of biologically active compounds [8,31,63]. Benbassat et al. evaluated the content of TPC and TFC in aqueous and hydroethanolic extracts from roots and concluded that 60% ethanol possesses the best extraction capacity, since these compounds are in the form of glycosides with higher polarity compared to the aglycone [39]. Sadighara et al. reported higher TFC in hydroethanolic extracts from white petals than pink petals [27]. Bahari et al. found that flavonoids are present in all organs of *A. officinalis* (leaves, roots, and flowers) but are more concentrated in flowers [52]. This trend was confirmed in all solvent groups in the present study.

In our study, alkaloids were obtained in minimal amounts, which is anticipated since marshmallow is an edible plant (Table 4). Only 9.7 ± 0.4 µgAE/L was extracted from the flowers using 70% ethanol. The leaf and root ethanolic extracts did not contain alkaloids. With NADES1, the values were 4.7 ± 0.2 µgAE/L (L1), 11.2 ± 0.5 µgAE/L (F1), and 2.5 ± 0.1 µgAE/L (R1). Alkaloids were not found in the extracts produced by NADES2. NADES1’s ability to obtain alkaloids from every part of the plant can be attributed to its strong acidity, which improves the solubility of the alkaloids in the solution. Different TalkC values were obtained by our research team in extracts from *M. neglecta* [50] and *M. sylvestris* [59]: the highest content of 21.7 ± 0.7 µgAE/L was measured in the *M. neglecta* root extract prepared by NADES1.

The antioxidant activity of marshmallow extracts was evaluated using three methods to explore different mechanisms of antioxidant defense: quenching ability against DPPH and ABTS radicals and metal ion chelating ability (by the FRAP method). The results are presented in Table 5. In the same solvent group, the flowers had the strongest DPPH and ABTS scavenging capacity: 85 ± 2 µmolTE/L and 89 ± 3% (F1), 72 ± 2 µmolTE/L and 73 ± 3% (F2), and 89 ± 2 µmolTE/L and 81 ± 3% (F3). The results obtained by the FRAP assay were not consistent with the trend. Better Fe-chelating ability was shown by the flower NADES extracts: 0.83 ± 0.05 mg Fe(II)/L (F1) and 8.09 ± 0.16 mg Fe(II)/L (F2). Among the ethanolic extracts, leaves showed the highest FRAP (3.95 ± 0.16 mg Fe(II)/L). Generally, the DPPH- and ABTS-radical scavenging ability of the root extracts was lower than that of leaf and flower extracts. Such a trend was confirmed in our previous studies on common mallow [59] and dwarf mallow green extracts [50].

To enhance the understanding of how specific groups of substances impact the antioxidant activity of the extracted samples, a correlation analysis was conducted (Table 6).

Intriguing correlations were established between the measured parameters. A positive correlation with high regression coefficient values was determined between DPPH radical scavenging capacity (RSC) and phenolic compound contents (TPC, TFC, and TCT), which is consistent with the positive correlation between RSC assessed by the DPPH method and TPC of mallow extracts documented by different researchers [59,64,65,66]. A positive correlation was also discovered between RSC and the antioxidant parameters FRAP and ABTS. The influence of TPC, TFC, and TCT had a strong impact on DPPH and ABTS but a very low effect on the FRAP assay (Table 5). Low and even negative correlations were identified for TAlkC and the other assessed parameters. Clearly, alkaloids do not play a role in oxidative stress resistance. This is consistent with the correlations between TAlkC and the antioxidant potential and constituents from our earlier research on *M. neglecta* [50] and *M. sylvestris* [59]. Marshmallow’s alkaloids have proven health benefits, e.g., gastro-protective activity [67]. Most of the reported alkaloids enhance mucus production as well [32].

### 2.2. Biological Activity

#### 2.2.1. Antibacterial Activity

Plants possess antimicrobial properties that are among their most significant features, enabling them to defend themselves against susceptible pathogenic microorganisms. This activity results from a variety of compounds that plants produce and release, including essential oils, phenols, flavonoids, saponins, terpenes, alkaloids, tannins, and glycosides, among others [68,69]. Numerous studies have indicated that the synergistic interactions of phytochemicals are crucial for the effective use of plant extracts as antimicrobial agents in fields such as biomedicine, agriculture, cosmetics, and the food industry [70]. The phytochemical analysis of *A. officinalis* showed that the plant contains various bioactive compounds, such as mucopolysaccharides, flavonoids, phenolic acids, tannins, coumarins, etc., with antimicrobial activity [12]. Also, *A. officinalis* has shown other pharmacological effects, both in vitro and in vivo, such as antitussive, anti-inflammatory, antioxidant, antiulcer, immunomodulatory, wound healing, infertility therapy, etc. [12,30]. As far as we know, there is no existing literature data on the antibacterial and antifungal properties of NADESs consisting of choline chloride, citric acid, and water, as well as the effectiveness of NADES extracts of *A. officinalis* against the bacteria and fungi studied in this investigation.

According to the results shown in Table 7, the solvent NADES1 demonstrated extremely strong antibacterial activity against all four tested bacterial strains. This activity was significantly higher not only in comparison with the other tested solvents—since NADES2 showed no antibacterial effect—but also when compared with the positive control, gentamicin. The remarkably high activity of NADES1 is most likely related to its much stronger acidity (pH = −0.17 ± 0.02) compared with NADES2 (pH = 2.63 ± 0.02) and ethanol (pH = 7.91 ± 0.01) (Table 4). These findings are consistent with the study by Gama et al., who investigated how gradual neutralization of wood vinegar acidity affects antibacterial activity. Their results indicated that increasing pH values were associated with a reduction in antibacterial effectiveness [70]. Similar research has also suggested that highly acidic environments can denature proteins in microbial cell walls, thereby disrupting cellular functions and inhibiting microbial growth [71]. The lower antibacterial activity of NADES1 against the Gram-negative bacteria *E. coli* and *P. aeruginosa*, compared with the Gram-positive bacteria *S. aureus* and *B. cereus*, may be attributed to the presence of an additional outer layer composed of lipopolysaccharides on the cell wall of Gram-negative bacteria. This structural barrier, which is absent in Gram-positive bacteria, reduces their susceptibility to the effects of NADESs [72].

In the present study, NADES1 extracts demonstrated the highest antibacterial potential against *E. coli*, followed by *S. aureus*, *P. aeruginosa*, and *B. cereus.* The experimental results mostly coincide with the findings concerning the antibacterial activity of NADES1 extracts from *M. neglecta* [50] and *M. sylvestris* [59]. Moreover, NADES1 extracts showed similar activity to ethanolic extracts against *S. aureus* and *P. aeruginosa*, lower activity against *B. cereus*, and higher activity against *E. coli* (against which leaf and flower ethanolic extracts of *A. officinalis* were not active), compared to the negative control. Generally, the differences in antibacterial activity between NADES1 and ethanolic extracts were relatively small. Leaf and flower NADES2 extracts exhibited higher activity against *S. aureus* than both NADES1 and ethanolic extracts, whereas the remaining NADES2 extracts were not active against the tested strains. The observed differences in antibacterial activity among the three types of extracts cannot be explained only by the data in Table 2, Table 3 and Table 4 regarding the content of biologically active compounds. Although 70% ethanol extracted considerably larger amounts of antibacterial compounds from the leaves and roots of *A. officinalis* and similar amounts from the flowers compared with NADES1 (Table 4), the antimicrobial effects did not correspond directly to these quantities.

In this experiment, NADES2 extracts contained lower levels of antibacterial compounds than both NADES1 and ethanolic extracts. Previous studies on *Malva sylvestris* and *Malva neglecta* have indicated that the high acidity of NADES1 extracts plays the most significant role in their antimicrobial activity, a trend that is also supported by the findings of the present study (Table 4) [50,59]. Jenny et al. also found that the antibacterial activity of extracts prepared with citric acid, such as NADES1 extracts, may partially result from the pH changes they induce [73]. Another study investigating deep eutectic solvent (DES) extracts of *A. officinalis* prepared with lactic acid reported antibacterial activity against *S. aureus*, *E. coli and P. aeruginosa* that was comparable to the results obtained for NADES1 extracts in the present study, with IZ values of 30, 32, and 35 mm, respectively. Moreover, the authors compared the antibacterial activity of lactic acid-based DES and methanolic and aqueous extracts of *A. officinalis* against the aforementioned bacteria. The authors observed significant antibacterial activity only for the DES extracts, while for aqueous and methanolic extracts, there was no activity [25]. The fact that NADES2 extracts displayed antibacterial activity only against *S. aureus* is likely associated with their relatively low acidity, as well as the low extraction yield of biologically active compounds compared with NADES1 and ethanolic extracts (Table 4). These results are consistent with previously reported findings regarding the antibacterial activity of NADES2 extracts from *M. sylvestris* and *M. neglecta*, with the sole exception of the comparatively high activity of leaf and flower extracts of *A. officinalis* against *S. aureus* [51,59]. As a whole, the leaf extracts demonstrated the highest antibacterial activity among all extracts studied. Against *S. aureus*, the NADES2 flower extract exhibited the largest IZ = 12.0 ± 0 mm compared to the negative control, with IZ = 6.0 ± 0 mm.

In this study, 70% ethanol showed very low antibacterial activity, which is consistent with the findings reported by Lim et al. [74]. Overall, the ethanolic extracts of *A. officinalis* demonstrated greater antibacterial activity against *B. cereus* compared to *S. aureus* and *P. aeruginosa*. Activity against *E. coli* was observed only in the root ethanolic extracts. However, they produced the largest IZ among the ethanolic extracts (11.3 mm). These findings are largely similar to the experimental results concerning the antibacterial potential of ethanolic extracts of *M. sylvestris* and *M. neglecta*. The only difference is that the root extracts of *A. officinalis* demonstrated moderate activity against *E. coli* (IZ = 11.3 mm), while ethanolic extracts of *M. sylvestris* and *M. neglecta* were not active against this bacterium [50,59]. The results of this study are consistent with other experiments that reported antibacterial effects of ethanolic extracts of *A. officinalis* against *S. aureus* and *B. cereus* [75,76]. Also, the values obtained partly coincide with the findings of Rezaei et al., which indicated that ethanolic extract of *A. officinalis* was active against *S. aureus* but not against *P. aeruginosa* and *E. coli* [26]. The reported differences could be attributed to climatic conditions, cultivation area, microbial strains, method of extract preparation, etc. [77].

#### 2.2.2. Antifungal Activity

Previous studies have shown that fungi are more resistant to organic acid-based NADESs compared to bacteria [51,59], which was also confirmed by the results of this experiment (Table 8). This is likely due to the structure of the fungal cell wall that is rich in chitin and glucans, which makes it harder for NADESs to penetrate [78]. Our expectations of pronounced antifungal activity focused on the NADES1 extracts from the flowers, where considerably higher levels of phenols, flavonoids, and alkaloids were detected compared to the leaves and roots (Table 4). However, contrary to our logic, the highest antifungal activity as a whole was demonstrated by NADES1 root extracts, which contained the least amount of biologically active compounds. The only possible reason for this could be the acidity of NADES1 root extracts (pH = 0.68 ± 0.02), which is higher than that of flower and leaf extracts, whose pH values were 0.96 ± 0.02 and 0.92 ± 0.02, respectively. These experimental results are similar to the data reported by Hassan et al., who found that 10% citric acid inhibited 20.16% of *Penicillium purpurogenum* and 17.71% of *A. flavus* growth [79]. Amphotericin B shows no activity against *P. chrysogenum*, *F. oxysporum*, and *A. ochraceus*, low activity against *A. niger*, low to moderate activity against *A. flavus* and *A. parasiticus*, and moderate activity against *A. carbonarius*. These findings are partially in line with the results of other authors who reported mostly low to moderate activity of amphotericin B against *Fusarium* spp., *Aspergillus* spp., and *Penicillium* spp. [80,81].

Overall, the activity of NADES1 is much higher than that of 70% ethanol. These results partly coincide with the data reported by Sequeira et al., who found that 70% ethanol has fungicidal properties against both *A. niger* and *P. chrysogenum* [82]. In the current study, neither NADES2 nor its extracts exhibited antifungal properties. These results are similar to the findings of other experiments regarding the antibacterial activity of NADES2 extracts from *M. sylvestris* and *M. neglecta* [50,59].

In this experiment, the pronounced trend of decreasing antifungal activity of NADES1 extracts compared to the NADES1 solvent was also observed in *M. sylvestris* and *M. neglecta* [50,59]. The sole exception was NADES1 root extracts against *P. chrysogenum* and NADES1 leaf and flower extracts against *A. ochraceus*. The most probable explanation for this trend is the decrease in acidity of NADES1 extracts compared to the pure solvent NADES1 (Table 4), which significantly influences the antifungal effect of NADES1 extracts [79].

In the present study, only root ethanolic extracts of *A. officinalis* exhibited very low antifungal activity against *A. carbonarius* with IZ = 8.0 mm. In all other cases, there was only trace activity or complete lack thereof (Table 8). Overall, higher antifungal activity was observed in ethanolic extracts of *M. sylvestris* and *M. neglecta* [50,59]. Other studies, however, have reported some activity against *F. oxysporum*, *Penicillium* spp. and *Aspergillus* spp. by ethanolic, aqueous, and dimethyl sulfoxide extracts of *A. officinalis* [83,84,85].

It is noteworthy that, regardless of the type of solvent, the highest antifungal activity overall was shown by the root extracts of *A. officinalis* compared to the leaf and flower extracts (Table 8). Such a tendency was also observed in the study of *M. sylvestris* and *M. neglecta* [50,59].

## 3. Materials and Methods

### 3.1. Plant Material

**Figure 5:**
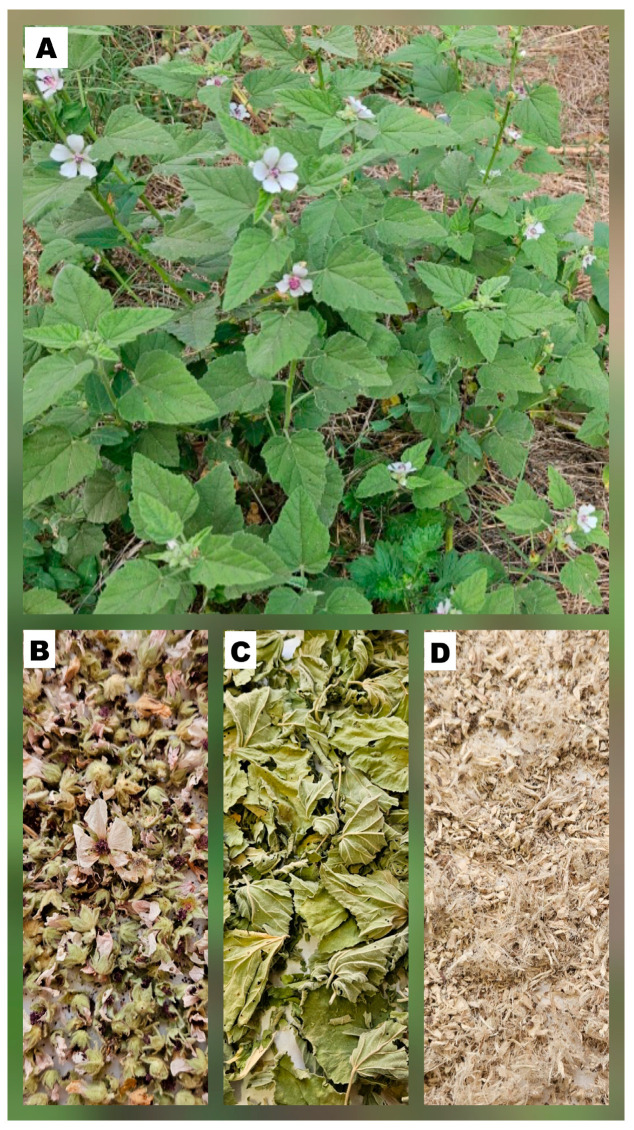
Plant material from *A. officinalis*: whole plant (**A**), flowers (**B**), leaves (**C**), and roots (**D**).

The materials investigated in this research were the leaves (L), flowers (F), and roots (R) of *A. officinalis* (Figure 5). The plant material was collected from a natural habitat in the village of Malka Vereya, Stara Zagora region, Mt. Sredna Gora, Bulgaria (42°41′7.5827″ N, 25°52′3.5375″ E). Aerial parts (leaves and flowers) were harvested during the peak flowering stage in August 2024, while the roots were collected at the end of the vegetative period in October 2024.

The botanical identification was confirmed, and voucher specimens from the studied population were deposited in the herbarium of the Agricultural University in Plovdiv City under the accession number SOA 063594. The collected material was air-dried in the shade at room temperature. Subsequently, the samples were ground using a mechanical grinder to a final powder size of less than 400 μm. Prior to analysis, the samples were stored in a dark and cool environment at temperatures of 16–18 °C.

### 3.2. Chemicals and Reagents

All reagents and organic solvents used in the chromatographic analyses were of LC/GC-MS-grade. Acetonitrile and methanol (Chromasolv^®^) were obtained from Honeywell Riedel-de Haen (Seelze, Germany). Formic acid was sourced from Sigma-Aldrich (Buchs, Switzerland). Ultrapure water was generated using a Smart2Pure 12 UV/UF system (Thermo Electron LED GmbH, Langenselbold, Germany). Authentic standards of caffeic acid (≥98%), rutin (≥95%), quercetin 3-glucoside (≥95%), kaempferol 3-rutinoside (≥95%), kaempferol 3-glucoside (≥95%) and tiliroside (≥95%) were obtained from PhytoLab GmbH & Co. KG (Vestenbergsgreuth, Germany). The experimental procedures, chemicals utilized, their mixtures, and concentrations are described in detail in the protocols of the applied methods in the following paragraph.

### 3.3. Microorganisms Studied

In this study, reference bacterial strains—*Bacillus cereus* ATCC 14579, *Staphylococcus aureus* ATCC 25923, *Escherichia coli* ATCC 25922, and *Pseudomonas aeruginosa* ATCC 27853—and reference fungal strains—*Fusarium oxysporum* NBIMCC 125, *Penicillium chrysogenum* NBIMCC 129, *Aspergillus niger* NBIMCC 3252, *Aspergillus carbonarius* NBIMCC 3391, *Aspergillus parasiticus* NBIMCC 2001, *Aspergillus flavus* NBIMCC 916, and *Aspergillus ochraceus* NBIMCC 2002—were included. The fungal strains were purchased from the National Bank for Industrial Microorganisms and Cell Cultures (NBIMCC), Bulgaria. All strains were stored at 0–4 °C.

### 3.4. Extraction Procedures

#### 3.4.1. Synthesis of Natural Deep Eutectic Solvents (NADESs)

The NADES systems were synthesized by heating and stirring the reaction mixture of HBA and HBD at a 1:1 molar ratio in round-bottom flasks. To optimize viscosity for room temperature applications, ultrapure water was incorporated into the mixture as a third component in quantities shown in Table 1. The protocol described by Memdueva et al. was followed [50]. The mixtures were subjected to continuous magnetic stirring at 80 °C for 8 h until a stable, transparent, and precipitate-free liquid was obtained. The resulting eutectic solvents were transferred to hermetically sealed glass containers and stored in the dark at room temperature prior to use.

#### 3.4.2. Extraction by NADESs

The plant sample was suspended in solvent at a ratio of 1.5:20, *w*/*v*. The extraction was carried out by mixing the suspension in a water bath for 60 min at 50 °C, followed by centrifugation for 35 min at 5300× *g* on a Heraeus Labofuge 200 centrifuge (Thermo Fisher Scientific, Waltham, MA, USA).

#### 3.4.3. Extraction by Classical Solvent

First, 70% *v*/*v* ethanol in water was chosen because hydroethanolic extracts showed better antioxidant capacity than pure ethanol [9]. The plant sample was suspended in the solvent at a ratio of 1.5:20, *w*/*v*. Then, the suspension was placed in an ultra-sonication bath for 30 min at 40 °C and 80 W/m^3^. This extraction technique was selected due to the quantity of the extraction of the target compounds [86].

To estimate the chromatographic profiling, the extracts prepared were lyophilized at −40 °C on a Biobase freeze dryer (Biobase Bioindustry Ltd., Jinan, China).

### 3.5. HPLC-PDA-MS Analysis

The methodology employed in this study followed the protocol described in our earlier work [79], with specific parameters detailed as follows: chromatographic analysis was achieved using an HPLC-PDA-ESI/MS system consisting of a Shimadzu LC-2040C 3D Nexera and a Shimadzu LCMS 2020 single quadrupole mass spectrometer (Shimadzu, Tokyo, Japan). Separation was performed on a Force C18 column (150 mm × 4.6 mm, 3 μm; Restek, Bellefonte, PA, USA) at 40 °C, with UV spectra recorded from 190 to 800 nm. The mobile phase comprises (A) 0.1% formic acid (FA) in water and (B) acetonitrile, following a gradient program of 5–30% B (0–30 min), 30–45% B (30–35 min), 45–95% B (35–37 min), 95% B isocratic (37–42 min), 95–5% B (42–43 min), and a 7 min re-equilibration. A flow rate of 0.4 mL/min and an injection volume of 5 μL were applied, with blank injections between samples for accuracy. The electrospray ionization parameters were optimized for negative ionization mode as follows: spray voltage: −3.50 kV; scan range: 100–1000 *m*/*z*; interface, desolvation line, and heat block temperatures: 350 °C, 250 °C, and 200 °C, respectively; nebulizing and drying gas flows were maintained at 1.5 L/min and 15 L/min. LabSolution software (Version 5.97 SP1, 2008–2019 Shimadzu Corporation, Kyoto, Japan) was used for data acquisition and processing. Lyophilized hydroethanolic extracts (F3, L3, and R3) were dissolved in methanol:water (70:30) at 2 mg/mL, while NADES extracts (F1, L1, R1 and F2, L2, R2) were diluted 1:20 in methanol:water:FA (50:50:0.1%) and ultrasonicated for 15 min. All samples were filtered through 0.22 μm PTFE syringe filters prior to analysis.

### 3.6. HPLC-HRMS/MS Analysis

Based on the methodology described in our earlier work [87], high-performance liquid chromatography coupled with high-resolution mass spectrometry (HPLC–HRMS/MS) analysis was performed using a Vanquish UHPLC coupled to a Q Exactive Plus Orbitrap^®^ (Thermo Fisher Scientific, Bremen, Germany). Separations were carried out on an Accucore C18 column (150 × 2.1 mm, 2.6 µm; Thermo Fisher Scientific, Bremen, Germany). The mobile phase consisted of solvent A (water containing 0.1% formic acid, *v*/*v*) and solvent B (acetonitrile), utilizing a gradient elution program of 5% B (0–2 min), 5–30% B (2–25 min), 30–95% B (25–30 min), 95% B (30–34 min), 95–5% B (34–35 min) and 5% B (35–40 min). The analysis was conducted at a constant flow rate of 0.3 mL/min with an injection volume of 3 μL. Data acquisition was performed in negative ion mode using a spray voltage of 2.90 kV and a capillary temperature of 320 °C. The sheath gas, auxiliary gas, and sweep gas flow rates were set to 30, 6, and 0 arbitrary units, respectively, and the S-Lens RF level was maintained at 50 V. Nitrogen was used both as the nebulizing gas and as the collision gas in the HCD cell. Full-scan MS spectra were acquired over an *m*/*z* range of 120–1200 at a resolution of 70,000, with an AGC target of 1 × 10^6^ and a maximum injection time of 80 m., while MS^2^ fragmentation was executed via data-dependent acquisition (Top 5). Key MS^2^ settings included a resolution of 17,500, AGC target of 1 × 10^5^, maximum injection time of 50 ms, and a 2.0 m/z isolation window and stepped normalized collision energies at 20, 40, and 70. Lyophilized hydroethanolic extracts (F3, L3, and R3) were prepared at 5000 mg/L in 50:50 water/methanol and diluted to a final concentration of 400 mg/L for analysis. Data acquisition was conducted using the Xcalibur software (version 4.2 SP1), and data analysis and processing were conducted using FreeStyle (version 1.5) and Compound Discoverer (version 3.3 SP3). All software packages are from Thermo Fisher Scientific.

### 3.7. GC-MS Analysis

For GC-MS profiling, the samples were prepared using a procedure that included derivatization. The protocol described by Dincheva et al. was followed, with some modifications [88]. Briefly, 50 mg of each lyophilized ethanol extract was mixed with 500 μL of methanol and 50 μL of 1 mg/mL ribitol (as internal standards), vortexed for 10 s, and incubated at 70 °C and 300 rpm for 30 min. After cooling to room temperature, 300 µL of distilled water and 500 μL of chloroform were added. The mixture was vortexed for 10 s and centrifuged at 13.000 rpm for 10 min at 22 °C. Then, 300 μL of the upper phase was vacuum-dried at 40 °C. To the dried residue, 100 μL of 20 mg/mL methoxyamine hydrochloride solution was added. The mixture was vortexed for 10 s and incubated at 70 °C and 300 rpm for 60 min. Then, 50 μL of N,O-Bis(trimethylsilyl)trifluoroacetamide (BSTFA) was added, and the mixture was heated again at 70 °C and 300 rpm for 30 min. After cooling, 300 μL chloroform was added, and 1.0 μL of the derivatized solution was injected into the GC-MS.

The GC-MS analysis was carried out on a 7890A gas chromatograph (Agilent Technologies, Santa Clara, CA, USA) with a 30 m × 0.32 mm (i.d.) HP-5ms capillary column (0.25 μm film thickness). The detection was conducted using a 5975C mass-selective detector (Agilent Technologies, Santa Clara, CA, USA). The chromatographic conditions were: 1.0 µL injection volume in split mode (10:1), carrier gas of helium at a flow rate of 1.0 mL/min, and an injector and detector temperature of 250 °C. The temperature program was as follows: start at 60 °C for 0 min, increase at a rate of 5 °C/min to 300 °C, and hold for 10 min. The mass spectrometer operated in electron impact (EI) mode at 70 eV, scanning a mass range of 50–550 *m*/*z*.

The compounds were identified by comparing their retention times and Kovats relative indices with those of standard substances [89] and mass spectral data from the NIST’08 library (National Institute of Standards and Technology, Gaithersburg, MD, USA) [90] and were quantified using the internal standard method in mg/g dm.

### 3.8. pH Values

The pH values of the crude extracts were measured on a pH meter, Consort 931 (Consort BVBA, Turnhout, Belgium).

### 3.9. Antioxidant Activity

The antioxidant activity of the investigated extracts was determined by three spectrophotometric methods with different mechanisms of action, following protocols described by Yaneva et al. [91]. These measurements were conducted on a UV-VIS spectrophotometer (Thermo Electron Scientific Instruments LLC, Madison, WI, USA).

#### 3.9.1. 2,2-Diphenyl-1-Picrylhydrazyl (DPPH) Method

An extract aliquot of 100 μL was added to 3.9 mL of 100 µM DPPH in methanol. After 30 min, the absorption at 517 nm was measured. A calibration was conducted using Trolox as a standard in the concentration range from 5 to 50 μmol/L (R^2^ = 0.9991). The results were expressed as µmol Trolox equivalent (TE) in 1 L extract.

#### 3.9.2. 2,2′-Azino-Bis(3-Ethylbenzothiazoline-6-Sulfonic Acid) (ABTS) Method

The solution of the radical ion was prepared by mixing a 7 mM ABTS aqua solution and 2.4 mM K_2_S_2_O_8_ at a ratio of 1:1 *v*/*v* at 20 °C in the dark for 24 h, followed by dilution with absolute EtOH to reach an absorbance of 0.700 at 734 nm. Then, 200 µL of the sample was added to 3.6 mL of the diluted reagent, and the absorbance was measured at the same wavelength. The results were calculated by Formula (1) and expressed as a percentage of radical inhibition:

(1) $$ABTS\;\left(\%\right)=\left(1-\frac{I_{x}}{I_{o}}\right)\times 100,$$

where I_0_ and I_x_ are the absorbance of the blank and of the sample, respectively.

#### 3.9.3. Ferric-Reducing Antioxidant Power (FRAP) Method

The FRAP reagent, prepared by mixing 100 mL of 300 mM sodium acetate buffer solution at pH 3.6, 10 mL of 10 mM TPZT, and 10 mL of 20 mM FeCl_3_, was added to 0.2 mL of the sample. The absorbance of the mixture was measured at 593 nm after 30 min incubation at 37 °C. Calibration using FeSO_4_ solution within the concentration range of 0.1–1.0 mM FeSO_4_ (R^2^ = 0.9981) was established. The results were expressed as mg equivalents Fe(II) in 1 L extract.

### 3.10. Total Phenolic Content (TPC)

TPC determination followed the procedure described by Yaneva et al. [91]. First, 1 mL sample was mixed with 5.0 mL of 1:10 *v*/*v* aqua-diluted Folin–Ciocalteu’s reagent. After the addition of 4 mL of 7.5% Na_2_CO_3,_ the mixture was left at room temperature for 60 min. The absorbance at 765 nm was measured against a blank. A calibration was established using gallic acid (Sigma-Aldrich, St. Louis, MO, USA) as a standard in a concentration range of 10–150 mg/L (R^2^ = 0.9992). The results were expressed as milligrams gallic acid equivalents (GAE) in 1 L extract.

### 3.11. Total Flavonoid Content (TFC)

TFC was determined by the aluminum trichloride method using catechin as reference material [77]. A mixture of 1 mL extract, 0.3 mL 5% NaNO_3_, and 4 mL deionized water was prepared. After 5 min, 0.3 mL of 10% AlCl_3_ was added, and after another 6 min, 2 mL of 1 M NaOH was added. After homogenization, the absorbance of the solution was measured at 510 nm. A calibration was established using standard solutions of catechin hydrate in concentrations from 10 to 150 mg/L (R^2^ = 0.9989). The results were expressed as milligrams catechin equivalent (CE) in 1 L extract.

### 3.12. Total Condensed Tannin Content (TCT)

TCT determination followed the experimental procedure described by Rebaya et al. [92]. A mixture of a 0.4 mL sample, 3 mL of 4% vanillin in methanol, and 1.5 mL of conc. HCl was prepared. After 15 min the absorbance was measured at 500 nm. A calibration was established using standard solutions of catechin hydrate in concentrations from 10 to 150 mg/L (R^2^ = 0.9995). The results were expressed as milligrams catechin equivalent (CE) in 1 L extract.

### 3.13. Determination of Total Anthocyanin Content (TAntC)

TAntC determination followed the experimental procedure described by Lee et al. [93]: The samples were mixed thoroughly with buffer pH 1.0 (0.025 M potassium chloride) and pH 4.5 (0.4 M sodium acetate buffer) at a 1:4 ratio. After 20 min incubation at room temperature, they were centrifuged at 4 °C and 12,000 rpm for 15 min. The absorbance of the supernatant was measured at 520 and 700 nm. TAntC was expressed as milligrams of cyanidin-3-glucoside equivalents (CGE) in 1 L extract after calculation by the following formulas:

(2) $$TAntC,\frac{mg}{L}=\frac{A\times D_{f}\times 1000}{\epsilon \times L}=\frac{A\times 449.2\times Df}{26.9}$$

(3) $$A={\left(A_{520}-A_{700}\right)}_{pH=1}-{\left(A_{520}-A_{700}\right)}_{pH=4.5}$$

where A_520_ and A_700_ are the absorbance values at 520 nm and 700 nm, respectively; 449.2 is the CGE molecular weight in g/mol; D_f_ is the dilution factor; ϵ is CGE molar absorptivity (26,900 L/mol/cm); and L is the cuvette length (1 cm).

### 3.14. Total Alkaloid Content (TAlkC)

TAlkC determination was conducted by a spectrophotometric method using bromocresol green (BCG) as the reagent [94]. An extract aliquot was suspended in 2N HCl and filtered. After adjustment of the pH of the aqueous layer to 7 by 0.1N NaOH, 5 mL of BCG solution (69.8 mg + 3 mL 2N NaOH + distilled water up to 1000 mL) and 5 mL phosphate buffer (pH 4.7) were added. The resulting colored complex was then sequentially extracted with 1, 2, 3, and 4 mL of chloroform. The volume of the collected chloroform extracts was adjusted to 10 mL, and the absorbance was read at 417 nm. A calibration was established using standard solutions of atropine in the concentration range from 40 to 120 mg/L (R^2^ = 0.9996), and the results were expressed as micrograms of atropine equivalent (AE) in 1 L extract.

### 3.15. Antimicrobial Activity

The antibacterial activity was evaluated using the agar well diffusion method, following the procedure outlined earlier by Velichkova et al. [95]. In summary, bacterial cultures incubated for 18–20 h on trypticase soy agar (TSA, Sigma-Aldrich, St. Louis, MO, USA) supplemented with 5% defibrinated sheep blood were utilized to prepare inocula in saline. The turbidity was adjusted to 0.5 McFarland standard (1.5 × 10^8^ CFU/mL) utilizing a Densilameter II (Erba Lachema, Brno, Czech Republic). Cation-adjusted Mueller–Hinton agar (Himedia, Maharashtra, India) was poured into Petri dishes to form an agar layer approximately 4 mm thick. The surface of the agar was inoculated by making three streaks with a sterile cotton swab dipped in the bacterial suspension while rotating the dish to guarantee uniform bacterial distribution. Wells with a diameter of 6 mm were subsequently created with a sterile cork borer and loaded with 100 μL of the examined extracts. Gentamicin (Himedia, Maharashtra, India) at a concentration of 10 μg/mL acted as the positive control, whereas the solvent served as the negative control. The plates were incubated aerobically at 37 °C for 24 h.

The antifungal activity of the extracts was assessed through the agar well diffusion method outlined by Velichkova et al. [95]. In summary, 72 h fungal cultures were cultivated on Potato Dextrose Agar (PDA, Himedia, Maharashtra, India). Approximately 20 mL of PDA was distributed into each Petri dish. Following solidification, the agar surface was inoculated by streaking three times with a sterile cotton swab previously dipped in the fungal suspension (1–2 × 10^4^ CFU/mL), while rotating the plate to ensure uniform distribution of the fungal cells. Wells measuring 6 mm in diameter were made with a sterile cork borer and loaded with 100 μL of the examined extracts. Amphotericin B (Sigma-Aldrich, Taufkirchen, Germany) at a concentration of 25 μg/mL acted as the positive control, whereas the solvent served as the negative control. The dishes were incubated at 26–28 °C for 3–5 days.

Antimicrobial activity was determined by measuring the inhibition zones (IZs) of microbial growth surrounding the wells containing the extracts. The inhibition zones were measured in millimeters and included the well diameter (6 mm). Antimicrobial activity was considered present when the inhibition zone was ≥8.0 mm. All experiments were performed in triplicate to ensure reproducibility of the findings, and the whole trial was performed under strict aseptic conditions.

### 3.16. Statistical Analysis

All results were presented as mean values ± standard deviation (±SD). The Pearson correlation analysis and linear regression were utilized to evaluate the relationships between the measured parameters. Statistical analysis of antimicrobial test data was conducted using one-way ANOVA followed by Fisher’s least significant difference (LSD) test with Statistica 10 (Statistica for Windows, StatSoft, Inc., Tulsa, OK, USA, 2010).

## 4. Conclusions

This study provides new insights into the phytochemical composition of *A. officinalis* and reveals a clear organ-dependent distribution of bioactive metabolites. The compounds detected by HPLC-MS included diverse hydroxycinnamic acid derivatives and flavonoid glycosides based on quercetin, kaempferol, and hypolaetin skeletons, several of which were identified or tentatively assigned for the first time in this species. GC-MS profiling of the ethanol extracts identified ten free amino acids, seven organic acids, and several mono- and disaccharides, as well as one oligosaccharide. Their concentrations varied across different parts of the plant depending on the specific metabolism of the respective organ. These findings expand the current knowledge of the phytochemical composition of *Althaea officinalis*.

Among the tested extracts, flower samples consistently demonstrated the highest antioxidant activity across all applied assays (DPPH—up to 89 µmol/L, FRAP—up to 89%, and ABTS—up to 8.09 mgFe(II)/L), indicating a higher accumulation of phenolic constituents in floral tissues. Ethanolic extracts generally exhibited higher total contents of phenols (up to 176 mgGAE/L), anthocyanins (up to 7.34 mgCE/L), and condensed tannins (up to 73 mgCGE/L), which likely contributed to their strong antioxidant performance. NADES1 extracts showed enhanced antibacterial potential compared to ethanolic extracts, likely related to their lower pH. NADES2 extracts exhibited limited antimicrobial effects, demonstrating activity solely in leaf and flower extracts against *Staphylococcus aureus*. The antifungal activity of all extracts was generally weak; NADES extracts showed no inhibitory effects, while only trace activity was observed in some ethanolic extracts.

Overall, the results highlight *A. officinalis* as an important source of natural antioxidants and show that natural deep eutectic solvents represent effective and sustainable alternatives to conventional solvents. These findings support the application of green extraction approaches for the valorization of medicinal and edible plants and encourage further exploration of NADES-based extraction strategies.

## Figures and Tables

**Table 1:** Extracts prepared from different plant organs of *A. officinalis*.

ID	Plant Organ	Solvent
L1	Leaf	NADES1 Choline chloride + Citric acid + Water (1:1 mol/mol) + 30% *w*/*w* Water
F1	Flower	Choline chloride + Citric acid + Water (1:1 mol/mol) + 30% *w*/*w* Water
R1	Root	Choline chloride + Citric acid + Water (1:1 mol/mol) + 30% *w*/*w* Water
L2	Leaf	NADES2 Choline chloride + Glycerol (1:1 mol/mol) + 30% *w*/*w* Water
F2	Flower	Choline chloride + Glycerol (1:1 mol/mol) + 30% *w*/*w* Water
R2	Root	Choline chloride + Glycerol (1:1 mol/mol) + 30% *w*/*w* Water
L3	Leaf	70% *v*/*v* Ethanol in water
F3	Flower	70% *v*/*v* Ethanol in water
R3	Root	70% *v*/*v* Ethanol in water

**Table 3:** Data from the GC-MS analysis of ethanolic extracts of leaves, flowers, and roots of *A. officinalis* (mean ± SD).

Metabolite	Leaves mg/g dm	Flowers mg/g dm	Roots mg/g dm
Free amino acids			
Glutamic acid	4.1 ± 0.9	3.2 ± 0.8	1.7 ± 0.5
Aspartic acid	3.6 ± 0.8	2.9 ± 0.7	1.4 ± 0.4
Alanine	2.2 ± 0.6	2.0 ± 0.5	0.9 ± 0.3
Serine	1.8 ± 0.5	1.7 ± 0.4	0.7 ± 0.2
Glycine	1.6 ± 0.4	1.5 ± 0.4	0.6 ± 0.2
Proline	2.4 ± 0.7	4.5 ± 1.1	0.8 ± 0.3
Asparagine	1.9 ± 0.5	2.3 ± 0.6	5.6 ± 1.4
Valine	1.2 ± 0.3	1.1 ± 0.3	0.5 ± 0.2
Leucine	1.0 ± 0.3	0.9 ± 0.2	0.4 ± 0.1
Phenylalanine	0.7 ± 0.2	0.6 ± 0.2	0.3 ± 0.1
Total	20.5	28.7	12.9
Organic acids			
Malic acid	10.8 ± 2.1	9.7 ± 2.0	5.6 ± 1.4
Citric acid	8.3 ± 1.9	7.4 ± 1.7	4.3 ± 1.1
Oxalic acid	6.1 ± 1.5	3.2 ± 0.9	2.0 ± 0.6
Succinic acid	2.6 ± 0.7	2.4 ± 0.6	1.6 ± 0.5
Fumaric acid	1.2 ± 0.4	1.1 ± 0.3	0.6 ± 0.2
Galacturonic acid	0.5 ± 0.1	nd *	10.7 ± 2.8
Glucuronic acid	nd	nd	6.4 ± 1.7
Total	29.5	23.8	17.5
Mono-, di-, and oligosaccharides			
Sucrose	45.2 ± 8.7	72.6 ± 12.5	31.8 ± 7.4
Glucose	32.4 ± 6.5	58.3 ± 10.9	21.6 ± 5.2
Fructose	28.9 ± 5.9	55.1 ± 10.4	19.7 ± 4.6
Maltose	9.6 ± 2.3	6.8 ± 1.9	nd
Raffinose	5.1 ± 1.4	9.2 ± 2.5	6.3 ± 1.8
Arabinose	nd	nd	12.4 ± 3.1
Galactose	nd	nd	11.1 ± 2.9
Rhamnose	nd	nd	5.0 ± 1.3
Total	121.2	201.9	108.0

* nd—not detected.

**Table 4:** pH values and overall concentration of biologically active substances in the crude extracts from *A. officinalis.*

ID	pH	TPC	TFC	TCT	TAntC	TAlkC
		mgGAE/L	mgCE/L	mgCE/L	mgCGE/L	µgAE/L
L1	0.92 ± 0.02	61 ± 5	31 ± 2	18 ± 2	nd *	4.7 ± 0.2
F1	0.96 ± 0.02	175 ± 7	109 ± 9	46 ± 6	0.29 ± 0.03	11.2 ± 0.5
R1	0.68 ± 0.02	19 ± 2	4.4 ± 0.5	17 ± 2	nd *	2.5 ± 0.1
NADES 1	−0.17 ± 0.02	-	-	-	-	-
L2	4.83 ± 0.02	30 ± 2	17 ± 2	13 ± 1	nd *	nd *
F2	4.85 ± 0.02	143 ± 5	58 ± 3	49 ± 5	nd *	nd *
R2	4.51 ± 0.02	11 ± 1	4.5 ± 0.6	nd *	nd *	nd *
NADES 2	2.63 ± 0.02	-	-	-	-	-
L3	6.17 ± 0.01	119 ± 3	76 ± 4	69 ± 6	1.04 ± 0.06	nd *
F3	5.82 ± 0.01	176 ± 4	79 ± 4	73 ± 6	7.34 ± 0.12	9.7 ± 0.4
R3	6.33 ± 0.01	32 ± 1	3.4 ± 0.5	16 ± 4	nd *	nd *
70% EtOH	7.91 ± 0.01	-	-	-	-	-

* nd—not detected.

**Table 5:** Antioxidant potential of the crude extracts from *A. officinalis*.

ID	DPPH	ABTS	FRAP
	µmol TE/L	%	mg Fe(II)/L
L1	71 ± 2	40 ± 2	0.34 ± 0.03
F1	85 ± 2	89 ± 3	0.83 ± 0.05
R1	39 ± 1	14 ± 1	0.57 ± 0.03
L2	69 ± 2	31 ± 2	5.44 ± 0.15
F2	72 ± 2	73 ± 3	8.09 ± 0.16
R2	45 ± 1	12 ± 2	2.52 ± 0.09
L3	76 ± 2	51 ± 3	3.95 ± 0.16
F3	89 ± 2	81 ± 3	2.37 ± 0.05
R3	42 ± 1	78 ± 2	1.82 ± 0.05

**Table 6:** Parameter correlation matrix of extracts from *A. officinalis.*

	TPC	TFC	TCT	TAntC	TAlkC	DPPH	ABTS	FRAP
TPC	1.0000	**0.9545** *	**0.8907**	0.5532	0.2749	**0.8640**	**0.7914**	0.1887
TFC		1.0000	**0.8430**	0.4248	0.4187	**0.8784**	**0.6987**	0.0856
TCT			1.0000	0.6485	−0.0157	**0.7648**	**0.6521**	−0.0654
TAntC				1.0000	−0.2213	0.5267	0.3890	−0.0654
TAlkC					1.0000	0.2296	0.1940	−0.5771
DPPH						1.0000	0.5926	0.2132
ABTS							1.0000	0.1162
FRAP								1.0000

* Values in bold are with r^2^ > 0.6500.

**Table 7:** Antibacterial activity of crude extracts of *A. officinalis* determined by measuring the diameter of inhibition zones (IZ) in mm (mean ± SD) *.

ID	Diameter of Inhibition Zones (mm)			
	*S. aureus*	*E. coli*	*P. aeruginosa*	*B. cereus*
L1	34.3 ± 1.5 ^ac^	32.7 ± 3.1 ^bc^	31.3 ± 1.2 ^ac^	30.7 ±1.2 ^ac^
F1	32.0 ± 0.0 ^ac^	31.3 ± 2.3 ^bb^	29.3 ± 1.2 ^ab^	32.0 ± 0.0 ^ab^
R1	33.3 ± 2.3 ^bc^	31.7 ± 3.9 ^bc^	29.3 ± 0.6 ^ab^	31.3 ±1.2 ^ab^
NADES1	30.0 ± 0.0 ^a^	27.0 ± 0.0 ^a^	28.3 ± 0.6 ^a^	30.0 ± 0.0 ^a^
L2	11.3 ± 1.2 ^ac^	- **	- **	- **
F2	12.0 ± 0.0 ^ac^	- **	- **	- **
R2	- **	- **	- **	- **
NADES2	6.0 ± 0 ^a^	6 ± 0 ^a^	6.0 ± 0 ^a^	6.0 ± 0 ^a^
L3	10.3 ± 1.2 ^ac^	- **	9.0 ± 1.7 ^ac^	10.7 ± 1.2 ^ac^
F3	8.7 ± 0.6 ^ab^	- **	9.0 ± 1.7 ^ac^	8.3 ± 1.5 ^ab^
R3	9.7 ± 0.6 ^ac^	11.3 ± 1.2 ^bc^	9.7 ± 0.6 ^ac^	9.3 ± 0.6 ^ac^
70% EtOH	7.0 ± 0 ^a^	7.0 ± 0.0 ^a^	7.0 ± 0 ^a^	6.7 ± 0.6 ^a^
Gentamicin	15.0 ± 0.0 ^ac^	11.0 ± 0.0 ^ac^	11.0 ± 0.0 ^ac^	15.0 ± 0.0 ^ac^

* Different letters in the columns denote significant differences between the inhibition zones of plant extracts and negative control (solvent) values according to one-way ANOVA and LSD tests (^a^ *p* ≤ 0.01; ^b^ *p* ≤ 0.05; ^c^ *p* > 0.05), ** no activity (IZ ≤ 6 mm).

**Table 8:** Antifungal activity of crude extracts of *A. officinalis* determined by measuring the diameter of inhibition zones in mm (mean ± SD) *.

ID	Diameter of Inhibition Zones (mm)						
	*P. chrysogenum*	*F. oxysporum*	*A. parasiticus*	*A. niger*	*A. flavus*	*A. carbonarius*	*A. ochraceus*
L1	13.0 ± 1.7 ^bc^	9.7 ± 0.6 ^ac^	- **	- **	11.0 ± 1.0 ^bc^	- **	10.0 ± 0.0 ^aa^
F1	14.0 ± 1.7 ^bc^	8.7 ± 0.6 ^ac^	- **	- **	11.7 ± 0.6 ^ab^	- **	10.0 ± 0.0 ^aa^
R1	15.7 ± 1.2 ^bb^	- **	8.0 ± 0.0 ^ac^	16.7 ± 0.6 ^bb^	11.7 ± 0.6 ^ab^	- **	9.7 ± 0.6 ^ab^
NADES1	15.3 ± 0.6 ^a^	10.0 ± 0.0 ^a^	9.0 ± 0.0 ^a^	17.7 ± 0.6 ^a^	12.0 ± 0.0 ^a^	6.0 ± 0.0 ^a^	10.0 ± 0.0 ^a^
L2	- **	- **	- **	- **	- **	- **	- **
F2	- **	- **	- **	- **	- **	- **	- **
R2	- **	- **	- **	- **	- **	- **	- **
NADES2	6.0 ± 0.0 ^a^	6.0 ± 0.0 ^a^	6.0 ± 0.0 ^a^	6.0 ± 0.0 ^a^	6.0 ± 0.0 ^a^	6.0 ± 0.0 ^a^	6.0 ± 0.0 ^a^
L3	8.3 ± 0.6 ^ab^	8.7 ± 0.6 ^ab^	7.3 ± 0.6 ^ab^	- **	7.7 ± 0.06 ^ac^	- **	8.3 ± 1.2 ^bc^
F3	8.0 ± 0.0 ^aa^	7.7 ± 0.6 ^ab^	7.3 ± 0.6 ^ab^	- **	7.0 ± 0.0 ^ab^	- **	7.7 ± 0.6 ^aa^
R3	8.7 ± 1.2 ^bb^	8.0 ± 0.0 ^ab^	7.7 ± 0.6 ^ab^	- **	7.7 ± 0.6 ^ac^	8.0 ± 0.0 ^aa^	7.7 ± 0.6 ^aa^
70% EtOH	8.0 ± 0.0 ^a^	7.7 ± 0.6 ^a^	7.0 ± 0.0 ^a^	6.0 ± 0.0 ^a^	6.0 ± 0.0 ^a^	6.0 ± 0.0 ^a^	7.7 ± 0.6 ^a^
Amphotericin B	6.0 ± 0.0 ^ab^	6.0 ± 0.0 ^ab^	11.0 ± 0.0 ^ac^	9.0 ± 0.0 ^ac^	11.5 ± 0.3 ^ac^	13.8 ± 0.3 ^ac^	6.0 ± 0.0 ^ab^

* Different letters in the columns denote significant differences between the inhibition zones of plant extracts and negative control (solvent) values according to one-way ANOVA and LSD tests (^a^ *p* ≤ 0.01; ^b^ *p* ≤ 0.05; ^c^ *p* > 0.05), ** no activity (IZ ≤ 6 mm).

## Data Availability

The raw data supporting the conclusions of this article will be made available by the authors on request.

## Abbreviations

The following abbreviations are used in this manuscript:

ABTS	2,2′-Azino-bis(3-ethylbenzothiazoline-6-sulfonic acid
BSTFA	N,O-Bis(trimethylsilyl)trifluoroacetamide
ChCl	Choline Chloride
CA	Citric Acid
DPPH	2,2-Diphenyl-1-picrylhydrazyl
FRAP	Ferric-Reducing Antioxidant Power
GC	Gas Chromatography
Gly	Glycerol
HBD	Hydrogen Bond Donor
HBA	Hydrogen Bond Acceptor
HPLC	High-Performance Liquid Chromatography
HRMS	High-Resolution Mass Spectrometry
IZ	Inhibition Zone
MS	Mass Spectrometer
NADES	Natural Deep Eutectic Solvents
PDA	Photo Diode Detector
TAlkC	Total Alkaloid Content
TAntC	Total Anthocyanin Content
TCT	Total Condensed Tannin Content
TFC	Total Flavonoid Content
TPC	Total Phenolic Content
SD	Standard Deviation
UV	Ultraviolet
UV-VIS	Ultraviolet–Visible
